# Books Received

**Published:** 1869-03-01

**Authors:** 


### BOOKS RECEIVED.

ON THE DYNAMICS, PRINCIPLES AND PHILOSOPHY OF ORGANIC LIFE: AN EFFORT TO OBTAIN DEFINITE CONCEPTIONS OF HOW DO MEDICINES PRODUCE THEIR EFFECTS? (Valedictory Address, delivered to the Muskingum County Medical Society, at its annual meeting held in the city of Zanesville, Onio, May 6, 1868.) By Z. C. MCELROY, M. D., Pres., of Zanesville, Ohio.

The essay with the foregoing title appears to be an attempt to establish some rational basis for the so-called science of therapeutics; an attempt whose success the entire medical world would welcome most joyfully.

It is certainly true, as the writer asserts, that we are more in need of a "more philosophical classification" of the mass of empirical facts now in existence, than of any additions thereto. This need he assnmes to have supplied in his theory, as set forth in the pamphlet before us, the essence of which appears to be a reduction of all organic processes into the two generic totals, "nutrition and oxydation," or constructive and destructive metamorphoses, and the operations of medicines to modifications of these two classes of phenomeua, by which they become promoters or retarders of nutrition or constructive metamorphosis, or of oxydation or destructive metamorphosis, respectively, a most attractive theory should it be sustained by a wider generalization, and more accurate analysis.

We think the prefatory quotation with which our author introduces his subject somewhat unfortunate, viz: "Since it is given us to know our own existence, and be conscious of our own individuality, we may rest assured that we have what in reality is a far less wonderful power, the capacity of comprehending all the conditions of our life." This *non sequitur* may not, possibly, transcend the scope of Professor Draper's "profound faith" in the intellect of man, but we think it entirely surpasses the present reach of that intellect.

Much credit should certainly be awarded to the author for the industry and ingenuity which he has displayed in the collection and arrangement of his data. We must, however, be permitted to express the opinion that he has fallen into that most common of all errors, to which the student of natural sciences, especially, is exposed, hasty generalization. We are inclined to think, too, that he has, sometimes, been led by the aptness of his analogies to forget that analogies, however striking, are yet not identities, and hence while they are frequently admirable as identities, they are as frequently worthless as proofs. Let us take, for instance, his favorite example, the electro-magnetic telegraph, in which he detects such striking resemblance in structure and function, normally, to the nervous system, that he arrives at the conclusion that the laws which govern, and the principles which underlie both are identical. This is certainly a step, and a long step in advance of the times, but it seems not unreasonable to follow the course pursued in determining organic analogies in comparative science, and apply pathological as well as physiological tests, and here we think the analogy ceases. The nervous system, when deranged functionally or structurally, manifests many and varied phenomena to which the electro-magnetic telegraph furnishes no analogies whatever, but in order to determine identities no variations are admissable under any aspcct.

In the brief space which can be spared in the columns of THE JOURNAL it is impossible to do more than to indicate the general character and scope of a work of this sort. We must leave to the individual reader the task of a more thorough analysis, with the assurance that, while we cannot admit many of the author's premises, and hence cannot justify the conclusions therefrom, and think, moreover, that in general the data are too restricted to sustain his inferences, there is much in the work to commend it to the thoughtful student, and we think it not at all improbable that the author has established an hypothesis which may ultimately constitute the basis for a true science of therapeutics, a consummation for the accomplishment of which he has our most hearty good wishes. W. H.

A HAND-BOOK OF UTERINE THERAPEUTICS AND OF DISEASES OF WOMEN. By Edward John Tilt, M. D., Member of the Royal College of Physicians, etc., etc. Second American edition thoroughly revised and amended. New York: *D. Appleton & Co.*, 90, 92 and 94 Grand Street, 1869. Pp. 345. Chicago: *W. B. Keen & Cook.*

This edition of a well-known and exceedingly useful book has been prepared by the author expressly for the American publishers. It would be a work of supererogation to say that this is now a standard treatise on the subject.

THE HEALTH-LIFT: Its Theory and Practice.

A beautifully-printed monograph of 38 pages, expounding the advantages of this particular mode of exercise, with a sketch of the principles upon which it is based. For sale at the city bookstores.

BRAITHWAITE'S RETROSPECT OF PRACTICAL MEDICINE AND SURGERY. Part LVIII—January. Uniform American edition. New York: *W. A. Townsend & Adams.* Chicago: *The Western News Company.* Paper. Pp. 304.

ARCHIVES OF OPHTHALMOLOGY AND OTOLOGY. Edited and published simultaneously in English and German. Prof. H. Knapp, M. D., in New York, and Prof. S. Moos, M. D., in Heidelberg. New York: *William Wood & Co.*

*Prospectus.*—The work is to be issued half-yearly, in parts of about three hundred octavo pages each, fully illustrated by fine engravings and superb chromo-lithographic plates. Subscription Price $7.00 *per annum* in advance.

